# Characterization of the *mrgRS *locus of the opportunistic pathogen *Burkholderia pseudomallei*: temperature regulates the expression of a two-component signal transduction system

**DOI:** 10.1186/1471-2180-6-70

**Published:** 2006-08-07

**Authors:** Magdy E Mahfouz, T Hilton Grayson, David AB Dance, Martyn L Gilpin

**Affiliations:** 1 Department of Biological and Geological Sciences, Faculty of Education, Kafr ElSheikh, Tanta University, Egypt; 2 School of Biological Sciences, University of Plymouth, England, UK; 3 Health Protection Agency South West, Derriford, Plymouth, England, UK

## Abstract

**Background:**

*Burkholderia pseudomallei *is a saprophyte in tropical environments and an opportunistic human pathogen. This versatility requires a sensing mechanism that allows the bacterium to respond rapidly to altered environmental conditions. We characterized a two-component signal transduction locus from *B. pseudomallei *204, *mrgR *and *mrgS*, encoding products with extensive homology with response regulators and histidine protein kinases of *Escherichia coli*, *Bordetella pertussis*, and *Vibrio cholerae*.

**Results:**

The locus was present and expressed in a variety of *B. pseudomallei *human and environmental isolates but was absent from other *Burkholderia *species, *B. cepacia*, *B. cocovenenans*, *B. plantarii*, *B. thailandensis*, *B. vandii*, and *B. vietnamiensis*. A 2128 bp sequence, including the full response regulator *mrgR*, but not the sensor kinase *mrgS*, was present in the *B. mallei *genome. Restriction fragment length polymorphism downstream from *mrgRS *showed two distinct groups were present among *B. pseudomallei *isolates. Our analysis of the open reading frames in this region of the genome revealed that transposase and bacteriophage activity may help explain this variation. MrgR and MrgS proteins were expressed in *B. pseudomallei *204 cultured at different pH, salinity and temperatures and the expression was substantially reduced at 25°C compared with 37°C or 42°C but was mostly unaffected by pH or salinity, although at 25°C and 0.15% NaCl a small increase in MrgR expression was observed at pH 5. MrgR was recognized by antibodies in convalescent sera pooled from melioidosis patients.

**Conclusion:**

The results suggest that *mrgRS *regulates an adaptive response to temperature that may be essential for pathogenesis, particularly during the initial phases of infection. *B. pseudomallei *and *B. mallei *are very closely related species that differ in their capacity to adapt to changing environmental conditions. Modifications in this region of the genome may assist our understanding of the reasons for this difference.

## Background

The saprophyte *Burkholderia pseudomallei *is an opportunistic pathogen that is capable of intracellular survival [1,2] and causes melioidosis, a frequently fatal disease of humans and animals, which can be difficult to diagnose [3]. Although the pathogen is mainly distributed in the soil and water of tropical regions, especially south-east Asia and northern Australia, it is highly adaptable, nutritionally versatile and able to survive and grow in a wide range of environments [4-8]. The disease encompasses a broad spectrum of clinical symptoms and outcomes, including long periods of latency up to 62 years [9], and affects the lives of many millions of people [10].

The pathogenesis of *B. pseudomallei *infections involves the expression of cell-associated components such as lipopolysaccharide, pili, extracellular polysaccharide, and flagella [3] as well as secreted factors including toxins [11], protease [12], siderophore [13] and phospholipase [14]. Although capsular polysaccharide has been shown to enhance the intracellular survival and virulence of the pathogen, the role of this and other factors in pathogenicity and host resistance has not been conclusively resolved [15-17]. It is possible that pathogenesis involves the expression of many genes that are regulated in response to complex environmental signals.

Many bacterial pathogens possess signal transduction systems that are able to elicit adaptive responses to environmental variations and consequently have an important role in regulating the expression of genes that are crucial for survival and infection [18]. The prevalence of two-component transduction systems in a wide variety of bacterial species has stimulated interest and some limited success in designing new classes of broad-spectrum antimicrobials that can block these signalling pathways [19,20]. The most attractive reason for targeting these systems, such as PhoP/PhoQ from *Salmonella typhimurium *and BvgA/BvgS from *Bordetella pertussis*, is that they control the expression of genes required for infectivity and virulence in pathogenic bacteria [21]. Bacterial two-component systems are typically composed of a transmembrane histidine protein kinase that serves as an environmental sensor and a cytoplasmic response regulator that uses reversible phosphorylation to regulate gene expression in response to changing environmental conditions [22,23].

Most, if not all, bacteria can respond and adapt to changes in environmental conditions, such as temperature, osmotic pressure and pH, by regulating gene expression [24,25]. Understanding how these conditions affect the expression of genes which can regulate an adaptive response is important for improving our understanding of opportunistic pathogens, such as *B. pseudomallei*, that are able to survive a wide range of environmental variations. In this study we report the identification of a two-component signal transduction system from *B. pseudomallei *encoding proteins most similar in structure to RcsB and RcsC that regulate capsule synthesis in *Escherichia coli*. We cloned and characterized the genes and examined their expression under conditions that *B. pseudomallei *may encounter during its life cycle. In order to more fully characterize this region of the *B. pseudomallei *genome we examined genetic variation downstream from the *mrgRS *locus in a variety of *B. pseudomallei *isolates and show that 2 distinct groups of isolates can be distinguished.

## Results and discussion

### Identification of genes encoding a two-component signal transduction system

**Figure 1:**
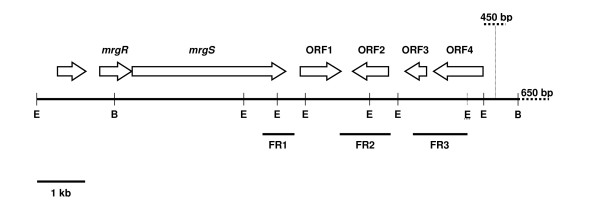
Map of the *mrgRS *locus of the *B. pseudomallei *genome (GenBank accession no. DQ418486). Open reading frames and direction of transcription are marked (arrows). See text for details of *mrgR *and *mrgS*, ORFs1-4, and oligonucleotide probes FR1-3. E = *Eco*RI site, B = *Bcl*I site. The positions of the additional *Eco*RI site that is described in the text and which is present in many *B. pseudomallei *isolates, and the 450 bp bacteriophage-like insertion and 650 bp transposase-like sequence that accompany this extra restriction site, are indicated by dotted lines.

A DNA sequence from the coding region of a two-component regulatory gene was amplified by PCR from *B. pseudomallei *204 chromosomal DNA, subcloned into pUC18, sequenced on both strands to confirm the identity, labelled and used to probe *B. pseudomallei *λZAP and λGEM-11 genomic DNA libraries. A λGT-11 genomic library constructed from *B. pseudomallei *strain 204 by Dr C. Davies, University of Plymouth was also screened. Approximately 4000, 5000 and 8000 recombinant phage plaques were screened from which 5, 3, and 2 positive plaques were isolated from the λGEM-11, λZAP Express, and λGT-11 libraries, respectively. Each was found to contain identical sequence information in the regions of overlap (Figure 1). This work was initiated and completed well before the *B. pseudomallei *genome sequence was published, and was not based on any genome sequence information.

Of the two open reading frames (ORFs) identified, one (666 bp) designated *mrgR *for **m**elioidosis agent **r**egulatory **g**ene **r**egulator, possesses a promoter but lacks an obvious transcriptional termination sequence after the stop codon. The translated product (23,860 Da) shares extensive similarity with a large family of response regulators, particularly the RcsB proteins of *Erwinia amylovora*, *E. coli*, and *Salmonella typhi*, and BvgA of *Bordetella pertussis *(28–31% identity). A helix-turn-helix DNA binding domain is located in the C-terminal portion (residues 158–215) which is a common feature of response regulators of the LuxR/FixJ family of cytoplasmic transcriptional regulators. In addition, the protein possesses conserved residues that characterize this family, including Asp-61 which is believed to be phosphorylated, and Asp-15, Asp-16, Thr-191, and Lys-202, which are believed to contribute to the acidic pocket for the phosphorylation site [18,26]. These features strongly suggest that MrgR should be assigned to the LuxR/FixJ family of transcriptional regulators that are located in the cytoplasm and are thought to bind to specific promoter sequences upstream of regulated genes.

The second ORF (3237 bp) was designated *mrgS *for **m**elioidosis agent **r**egulatory **g**ene **s**ensor and is transcribed in the same direction as *mrgR*. The initiation codon of *mrgS *overlaps by a single nucleotide with the termination codon of *mrgR *i.e. TG**A**TG. This feature has been described for genes encoding two-component regulator systems from different bacterial species such as *ompR/envZ *of *E. coli *[27] and *irlRS *operon of *B. pseudomallei *[28]. Eight nucleotides upstream from the ATG start codon and in the distal portion of *mrgR*, there is a potential S-D sequence (AAGGA) and 17 bp downstream from the stop codon lies a 36 bp GC-rich inverted repeat (ΔG = -26 kcal) which could act as a strong transcriptional terminator for *mrgS*. The absence of secondary structure around the stop codon of *mrgR *suggests that transcription does not terminate at that point and the lack of an obvious promoter region for *mrgS *suggests that transcription may be controlled from the *mrgR *promoter and that both ORFs are transcribed as a polycistronic unit.

The translated product of *mrgS *(118,267 Da) shares homology with a family of two-component histidine kinases that function by sensing environmental stimuli, particularly RcsC of *E. coli *and also sensor kinases of *S. typhi *and *Vibrio cholerae *(18–22% identity). RcsC is the sensor regulator of capsule synthesis in *E. coli *and many other species, including *E. amylovora*,*P. mirabilis *and *S. typhi *[29]. MrgS possesses many of the features common to this family of sensor proteins including two strongly hydrophobic transmembrane sequences located in the N-terminal portion of the protein between residues 29–49 and 322–342, and five blocks of conserved functional subdomains located in the C-terminal portion of the protein including His-503 that is the proposed site of phosphorylation, and the N, G_1 _(DTGVG), F, and G_2 _(GTGLG) boxes at positions 620, 647–651, 661 and 677–681, respectively [30]. These motifs are presumed to form a nucleotide-binding surface within the active site of the molecule. In addition, several conserved Asp residues are present in MrgS, at positions 860, 898, 903, 910 and 949, and may contribute to the additional response regulator domain that is a characteristic of hybrid histidine kinases [31]. Like BvgS of *B. pertussis*, MrgS is a member of a family of complex sensor proteins, the hybrid kinases, which contain multiple cytoplasmic domains that are believed to participate in a phosphorylation cascade in signal transduction. The presence of two hydrophobic transmembrane domains separated by a large loop of 273 amino acids which may act as a periplasmic environmental monitor is appropriate for a sensor protein that lends itself to signal transduction [18].

Because the deduced amino acid sequences of *mrgR *and *mrgS *are most similar to the sequences of genes encoding known response regulators and sensors, it has been inferred that these two genes constitute a two-component signal transduction system. Since the completion of our work the *B. pseudomallei *genome sequence has been published [32] and *mrgR *and *mrgS *are now known to correspond to locus tags BPSL1633 and BPSL1634, respectively, in the genome sequence of *B. pseudomallei *K96243.

### The *mrgRS *locus is present in *B. pseudomallei *but not other *Burkholderia *species

**Figure 2:**
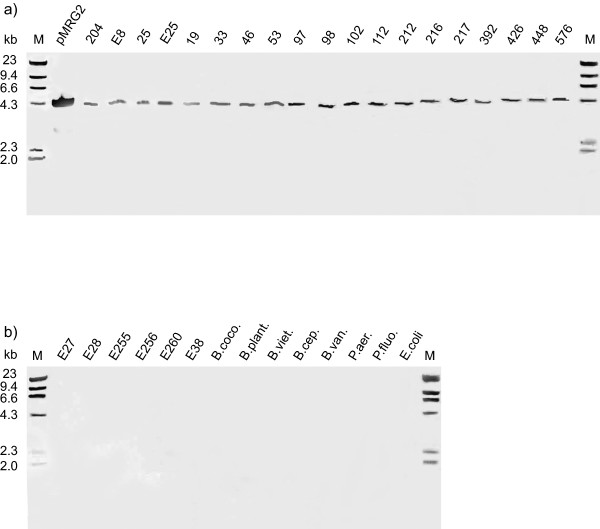
Southern blot hybridisation of *Eco*RI-digested genomic DNA from different bacterial species using a digoxigenin-labeled oligonucleotide probe for *mrgRS*: a) *B. pseudomallei *isolates, pMRG2 contains a 4.3 kb *Eco*RI fragment of *B. pseudomallei *genomic DNA, spanning most of the *mrgRS *locus, inserted in pUC18 and digested with *Eco*RI b) *B. thailandensis*, other *Burkholderia *species, including *B. cocovenenans *(B. coco.), *B. plantarii *(B. plant.), *B. vietnamiensis *(B. viet.), *B. cepacia *(B. cep.) and *B. vandii *(B. van.), *Pseudomonas aeruginosa *(P. aer.), *Pseudomonas fluorescens *(P. fluo.) and *E. coli*. Lane M, λ-HindIII markers with molecular sizes (in kilobases) indicated on the left. See Table 1 for isolate details. All of the samples in the figure were separated on the same single agarose gel, transferred to the same single nylon membrane, and hybridized in the same single hybridization tube using the same labeled probe. The image of the resulting single Southern blot is reproduced in the figure as 2 panels, a) and b), for convenience and clarity and therefore pMRG2 represents a positive control for all samples.

Southern hybridization analysis of genomic DNA extracted from 19 isolates of *B. pseudomallei *derived from environmental and clinical samples from different areas of the world (Table 1), showed that all of the isolates possessed a single *Eco*RI fragment of ~4.3 kb in size (Figure 2a) corresponding to the *Eco*RI fragment spanning most of the *mrgRS *locus. The locus was not detected in genomic DNA from *E. coli*, *Pseudomonas aeruginosa*, *Pseudomonas fluorescens*, or other *Burkholderia *species including the closely related *Burkholderia thailandensis *(Figure 2b). We compared the *mrgRS *locus from *B. pseudomallei *204 with the completed genome sequences for *B. pseudomallei *isolates K96243 and 1710b and with the unfinished sequences of a further 8 strains that are available on GenBank and found that there is some variation amongst the different strains within this region of the genome. Although the *mrgR *reading frame is preserved there are modifications within the 5' region upstream from the *mrgR *start codon that may have implications for the expression of the gene. In some strains there are also modifications within the coding region of *mrgS*, including a single nucleotide addition (nt 3051) that changes the reading frame and adds 4 additional amino acids to the last 61 residues of the C-terminus of MrgS and which may have functional consequences. A search of the genome sequences of 8 strains of the obligate pathogen *Burkholderia mallei *which are available on GenBank, revealed that 2128 bp encompassing the entire *mrgR *gene sequence and including 1319 bp upstream of the *mrgR *start codon, is highly conserved in 4 of the 8 strains of this organism (98% identity over 2128 bp). The full *mrgR *gene sequence shares 100% identity with *B. mallei*. Apart from the initial 144 bp downstream from the start codon, the *mrgS *sequence is absent from *B. mallei *and this feature may contribute to its inability to adapt to conditions outside a suitable host, in contrast with *B. pseudomallei *[33,34].

**Table 1:** Bacterial strains used in this study

Strain	Strain origin	Sample	Geographical origin	Date	Source
***Burkholderia pseudomallei***					
E8	Environment	Soil	NE Thailand	1990	1
19	Environment	Soil	Singapore	1991	1
22	Environment	Soil	Burkina Faso	1973	2
25	Environment	Soil	Madagascar	1977	1
E25	Environment	Soil	Thailand	-	1
33	Environment	Manure	France	1976	1
46	Human	Blood	NE Thailand	1988	1
53	Human	Urine	NE Thailand	1987	1
97	Environment	Soil	Australia	-	1
98	Environment	Soil	Australia	-	1
102	Environment	Soil	Australia	-	1
112	Human	Multiple	NE Thailand	1992	1
204	Human	Blood	Thailand	-	1
212	Environment	Soil	NE Thailand	1990	1
216	Environment	Soil	NE Thailand	1990	1
217	Environment	Soil (wet)	NE Thailand	1990	1
392	Human	Pus	NE Thailand	1989	1
426	Environment	Soil	Vietnam	-	1
448	Environment	Soil	Vietnam	-	1
576	Human	Blood	Thailand	-	1
Hainan 1 (H1)	Human	Abscess	China	1996	2
Hainan 2D (H2D)	Human	Abscess	China	1996	2
Hainan 55 (H55)	Human	Abscess	China	1996	2
Hainan 706 (H706)	Human	Abscess	China	1996	2
					
***Burkholderia thailandensis***					
E27	Environment	Soil	Thailand	-	2
E38	Environment	Soil	Thailand	-	2
E82	Environment	Soil	NE Thailand	1990	2
E255	Environment	Soil	NE Thailand	-	2
E256	Environment	Soil	Thailand	-	2
E260	Environment	Soil	Central Thailand	1993	2
					
**Other *Burkholderia *species**					
*B. cocovenenans *LMG11626					2
*B. plantarii *LMG10908					2
*B. vietnamiensis *LMG6998					2
*B. cepacia *IIIa non-epidemic					2
*B. vandii *LMG10620					2
					
**Other species**					
*Pseudomonas aeruginosa*					3
*Pseudomonas fluorescens*					3
*Escherichia coli *DH5α					3

1: David Dance HPA South West, Derriford, Plymouth; 2: Ty Pitt, Centre for Infections, HPA, Colindale, London; 3: University of Plymouth culture collection

### MrgR and MrgS are present in *B. pseudomallei *cell lysates but not culture supernatant

**Figure 3:**
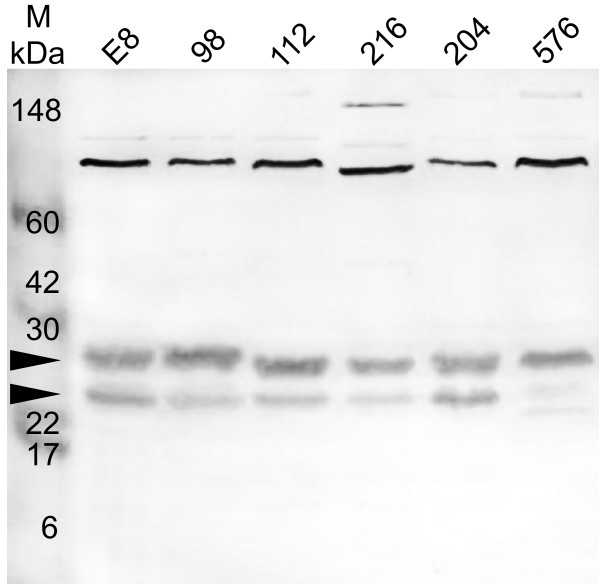
Western blot probed with an affinity purified antibody, anti-MrgR, showing the detection of MrgR in whole cell lysates from 6 isolates of *B. pseudomallei *grown at 37°C. The isolate number is indicated above each lane. Lane M: molecular weight markers indicated in kilodaltons (kDa). See Table 1 for isolate details. Arrows indicate the expected position of the 24 kDa MrgR protein and slightly larger phosphorylated forms of MrgR. See text for details.

**Figure 4:**
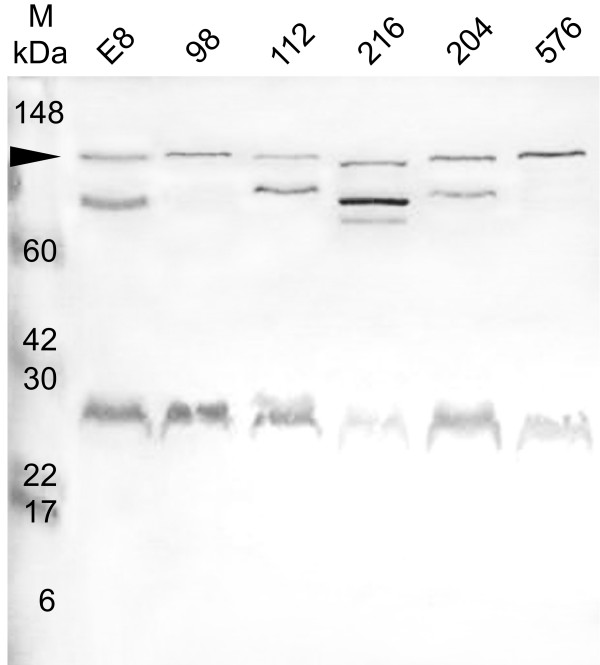
Western blot probed with an affinity purified antibody, anti-MrgS, showing MrgS in cell lysates from 6 isolates of *B. pseudomallei *grown at 37°C. The isolate number is indicated above each lane. Lane M: molecular weight markers indicated in kilodaltons (kDa). See Table 1 for isolate details. Arrow indicates the expected position of the 118 kDa MrgS protein. Smaller bands may represent processed forms of MrgS. See text for details.

Strains of *B. pseudomallei *representing isolates from clinical (112, 204, 576) and environmental (E8, 98, 216) samples were cultured at 37°C and whole cell lysates and concentrated culture supernatants were prepared, proteins were separated by SDS-PAGE, transferred to nitrocellulose and probed with antibodies recognising MrgR and MrgS. Anti-MrgR and anti-MrgS each recognized more than one band on Western blots of whole cell lysates of all strains. In all strains, anti-MrgR recognized 3 major bands close to the expected size of MrgR, 24–28 kDa, as well as a strong band of ~80 kDa (Figure 3). Members of the response regulator protein family are known to be phosphorylated at specific residues [26] and because MrgR possesses the same features as these proteins we propose that the 24–28 kDa bands may represent different phosphorylation states of the MrgR protein. In some strains, bands of ~90 and/or ~150 kDa were faintly visible. Anti-MrgS strongly stained a band of ~115 kDa, close to the predicted size of MrgS, and another band of ~30 kDa (Figure 4). In some strains a band of ~90–100 kDa was also visible. Preincubating anti-MrgR and anti-MrgS with their cognate immunising peptide, as described in the antibody specificity data supplement (see Additional file 1), abolished all staining. Hence additional bands possess the same epitope and may represent either breakdown products of the 118 kDa MrgS protein or, in the case of MrgR, an additional antigenically-related 80 kDa protein. MrgR and MrgS were not detected in concentrated broth culture supernatant (result not shown) supporting the suggestion that MrgR and MrgS are not secreted proteins.

### Convalescent sera from melioidosis patients reacts with MrgR

**Figure 5:**
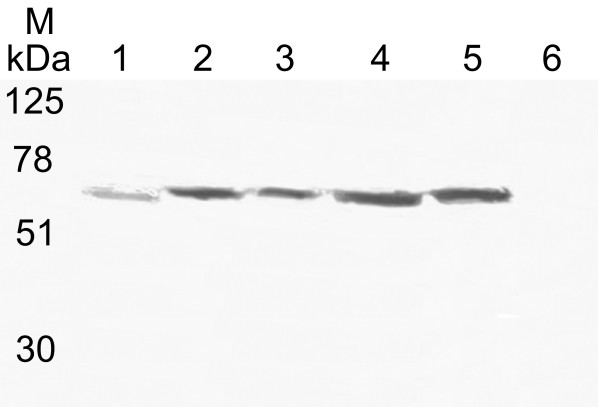
Western blot showing the recognition of MBP-MrgR fusion protein by antibodies in convalescent sera pooled from 6 melioidosis patients. Lane 1: Uninduced cells, lane 2: cells induced for 3 h with IPTG, lane 3: amylose resin purified cell extract, lane 4: crude cell extract, and lane 5: insoluble matter, lane 6: purified maltose binding protein. Lane M: molecular weight markers indicated in kilodaltons (kDa).

Western blots of whole cell lysates from 6 isolates of *B. pseudomallei *that were probed with convalescent sera pooled from 6 melioidosis patients (supplied by Vanaporn Wuthiekanun, Wellcome Trust-Oxford University-Mahidol University Tropical Medicine Research Programme, Bangkok, Thailand) demonstrated strong antibody recognition of multiple components in all isolates (see Additional file 4). Furthermore, Western blots of cell lysates of *E. coli *expressing MBP-MrgR that were probed with the same sera stained a band of ~60 kDa that was present in soluble, insoluble, and amylose resin purified extracts from bacteria that had been induced with IPTG (Figure 5). A low level of recognition was evident in uninduced cell extracts, similar to that observed when Western blots of the same cell extracts were probed with anti-MrgR. The convalescent sera did not recognize purified MBP, MBP-MrgS or any other *E. coli *components and no recognition of MBP-MrgR was seen when Western blots of the same extracts were probed with serum from an individual not previously exposed to *B. pseudomallei *(data not shown). This raises the possibility that MrgR may be expressed by *B. pseudomallei *during melioidosis infection although it is unclear how a regulatory protein that is not apparently secreted *in vitro *would be exposed to the host immune system and the presence of cross reactive epitopes on related proteins can not be excluded.

### Expression of MrgR and MrgS proteins under different growth conditions

*B. pseudomallei *204, from which the *mrgRS *locus was cloned and characterized, was cultured under a variety of different conditions. A range of temperatures, pH and salinities were chosen to reflect a variety of natural and host environments to which *B. pseudomallei *might be exposed within the tropics [6]. Although *B. pseudomallei *can grow over a wide range of variation in these parameters, we obtained the highest yields of protein at 42°C, pH 6.8 and 0.5% NaCl. Substantial yields of protein were also obtained at either 37 or 42°C, pH 5 and 0.15% NaCl, and the lowest yields were recorded at pH 8. These observations support *in vitro *and *in vivo *studies showing that *B. pseudomallei *readily adapts to acidic environments [7,35,36] and has an optimum growth temperature *in vitro *of 37–42°C [6]. Birds are considered to be relatively resistant to *B. pseudomallei *infection [37] but the strong growth at 42°C of all of the isolates of *B. pseudomallei *raises the possibility that birds might act as a reservoir of *B. pseudomallei *and pose a risk for the transmission of active melioidosis to other species [38].

**Figure 6:**
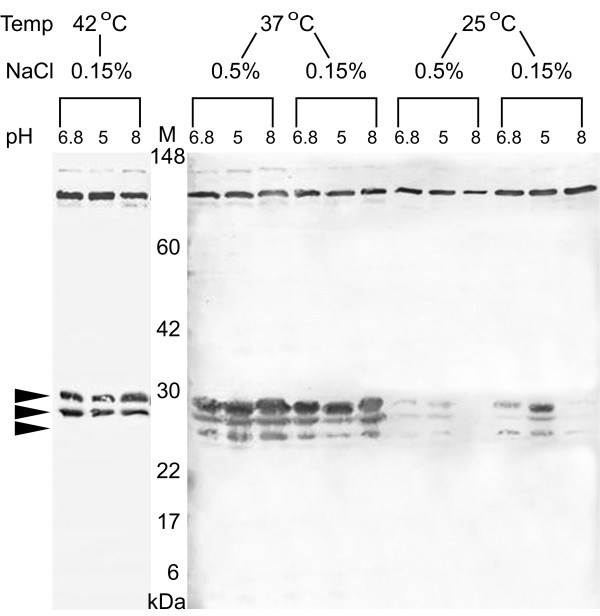
Expression of MrgR in *B. pseudomallei *204 cultured under different combinations of temperature, pH and NaCl concentration. Western blots of whole cell lysates were probed with anti-MrgR. The identity of each lane is indicated at the top. Lane M: molecular weight markers indicated in kilodaltons (kDa). Arrows indicate the expected position of the 24 kDa MrgR protein and slightly larger phosphorylated forms of MrgR.

**Figure 7:**
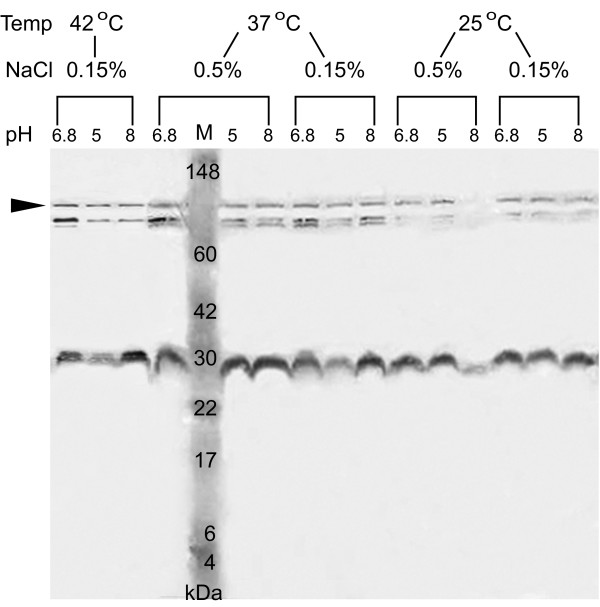
Expression of MrgS in *B. pseudomallei *204 cultured under different combinations of temperature, pH and NaCl concentration. Western blots of whole cell lysates were probed with anti-MrgS. The identity of each lane is indicated at the top. Lane M: molecular weight markers indicated in kilodaltons (kDa). Arrow indicates the expected position of the 118 kDa MrgS protein.

The expression of the 24–28 kDa MrgR bands was substantially reduced when *B. pseudomallei *204 was cultured at 25°C compared with 37 or 42°C but was mostly unaffected by different pH or NaCl concentration, although at 25°C and 0.15% NaCl a small increase in expression was observed at pH 5 (Figure 6). This was particularly evident at 42°C, where the lower molecular weight band that we believe represents the dephosphorylated form of MrgR is absent, demonstrating that at higher temperatures the gene is co-ordinately up-regulated. It is possible that at least some of the observed effects could be explained by differences in gene expression at different growth stages, but because equivalent quantities of protein from each sample were used in these studies and the expression of the 80 kDa immunoreactive band was mostly unaffected by pH, salt concentration or temperature then this possibility seems unlikely. A similar response was observed for MrgS expression (Figure 7).

### Cloning and sequence analysis of *mrgRS *downstream flanking region

Four ORFs, referred to here as ORFs1-4, were identified downstream from the *mrgRS *locus (Figure 1) and the conceptual translations of each ORF were analysed. The sequence and arrangement of the ORFs is available on GenBank (accession no. DQ418486). We cloned, sequenced and analyzed the genes well before the completion of the *B. pseudomallei *genome sequence, which is now known to correspond to the region of the *B. pseudomallei *K96243 genome sequence containing locus tags BPSL1635-1637. These locus tags do not exactly match ORF2 and ORF3 that we have identified. Indeed, there are differences in the nucleotide sequences and also in the gene and protein annotations for this region of the two completed genome sequences, *B. pseudomallei *K96243 and 1710b that are listed on GenBank. The strong transcriptional termination sequence following the *mrgS *gene suggests that the downstream sequences are transcribed separately. ORF1 (BPSL1635) is immediately downstream of *mrgS *and transcribed in the same direction and the predicted protein (31,527 kDa) is similar to sensor transduction proteins containing EAL (GluAlaLeu) domains, such as the oxygen sensing protein of *E. coli *O157:H7 (31% identity) and BvgR of *B. pertussis *(25% identity). EAL domains are found in a wide variety of bacterial signalling proteins where they can act to stimulate degradation of the second messenger, cyclic di-GMP, and may function as a diguanylate phosphodiesterase [39]. Enzymatically active and inactive forms have been described [39] and the variation in EAL domain sequences may reflect the convergent evolution of successful structure-function motifs rather than the usual primary sequence similarity. This structure is conserved in the conceptual translation of ORF1. Although the precise functions of many of these proteins are unknown, their open reading frames are within or are tightly linked to operons with well-defined functions. For example, *bvgR *is required for regulation of all known *bvg*-repressed genes in *B. pertussis*, and is located on a separate regulatory unit immediately downstream from the *bvgAS *locus encoding a two-component regulatory system that upregulates virulence gene expression in *B. pertussis *[40]. The similarities between the genomic organisation in *bvgAS*-*bvgR *and *mrgRS*-ORF1 together with the fact that both BvgAS and MrgRS are members of the two-component signal transduction family allows for the possibility that ORF1 may also act to repress the *mrgRS *locus.

Further downstream and in the reverse direction of the complementary strand three open reading frames were identified, ORF2, ORF3, and ORF4. For ORF2, the predicted protein (28,251 kDa) is similar to the arginine/serine rich regions of functionally diverse proteins that are important for binding RNA and DNA and for protein-protein interactions [41], such as LigA of *Burkholderia cenocepacia *(33% identity). The conceptual translation of ORF3 (16,856 kDa) is similar to a variety of transmembrane-transport proteins, such as the ABC thiamine transporter of *Roseovarius nubinhibens *(31% identity) and capsular polysaccharide export protein of *Rhodobacter sphaeroides *(28% identity), and possesses 2 transmembrane helices (TopPRED2 scores 0.71 and 0.88). For ORF4 (BPSL1637), the predicted protein (39,493 kDa) has a lipase (class 3) domain spanning residues 103–252 and including the lipase serine active site [42], GHSLG, located at positions 187–191. This protein lacks the features usually associated with a signal peptide, although this is not an essential component for secretion [43], and is strongly predicted to be secreted by non-classical mechanisms, SecP score = 0.933881 (*B. pseudomallei *isolate 204) and 0.930609 (*B. pseudomallei *isolate 112) [44]. *B. pseudomallei *has been shown to utilize a wide variety of lipid substrates [45,46] and to confirm the presence of an extracellular lipase concentrated culture supernatants were prepared from all isolates of *B. pseudomallei *(Table 1). Lipase activity was determined to be present in all samples using the fluorogenic substrate 4-methylumbelliferyl oleate and so it is possible that this gene could be expressed, secreted and contribute to this lipase activity, either alone or in concert with other molecules.

The full response regulator gene *mrgR *is present in the genome of many *B. mallei *isolates but *mrgS *and the downstream flanking region are absent. Instead, ORFs immediately downstream of the *mrgR *homolog in *B. mallei *have strong homology to penicillin amidase, carbamoyl transferase and transposase and their presence provides circumstantial evidence that transposition may partly explain the absence of this region in *B. mallei*.

### Restriction fragment length analysis of the *mrgRS *downstream flanking region

Three oligonucleotide probes, FR1, FR2 and FR3, covering almost 5 kb of the *B. pseudomallei *genome downstream from *mrgRS *(Figure 1) were used to investigate *Eco*RI digests of genomic DNA from different *B. pseudomallei *strains as well as *B. thailandensis *(Table 1). No signal was obtained with any of the probes in any of the *B. thailandensis *isolates. Furthermore, this region is not present in the recently completed *B. thailandensis *E264 genome sequence [33] that is available on GenBank.

**Figure 8:**
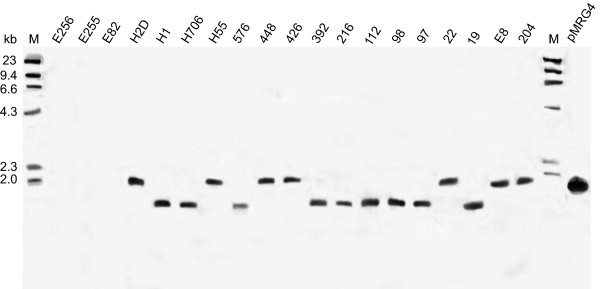
Southern blot hybridisation of *Eco*RI-digested genomic DNA from isolates of *B. pseudomallei *and *B. thailandensis *using oligonucleotide probe FR3. Lane M, λ-HindIII markers with molecular sizes (in kilobases) indicated on the left. Lane pMRG4 contains an 8.4 kb *Bcl*I fragment of the *B. pseudomallei *204 genome spanning most of the *mrgRS *locus and 5 kb downstream, inserted in the phagemid pBK-CMV which was excised from λZAP Express and digested with *Eco*RI. See Table 1 for isolate details.

The FR1 probe spans the distal end of *mrgS *and 285 nucleotides further downstream from the *mrgS *stop codon including an *Eco*RI site, and was expected to hybridise two *Eco*RI genomic DNA fragments of 684 and 592 bp. These two bands were present in most isolates of *B. pseudomallei *except 392 and H55 which possessed the 592 bp fragment only (data not shown). In isolate 392 this band is very strongly stained and may represent a doublet. In addition, H55 possessed an additional unique band of approximately 4.5 kb. There were no obvious variations in fragment size between isolates in the adjoining region that was probed with FR2 (data not shown). However, the region probed with FR3, which lacked an internal *Eco*RI site, revealed two distinct patterns between *B. pseudomallei *isolates. One pattern indicated the presence of a single band of ~1781 bp in seven isolates, while the other pattern demonstrated a band of ~1421 bp in nine isolates (Figure 8). To determine the reasons for the difference, we sequenced this part of the genome of isolates possessing each size of *Eco*RI fragment.

Alignment of 1472 nucleotides covering the same region of the genomes of isolates 112 and 204 revealed 19 base substitutions including one substitution of "A" for "G" at position 1119 which introduces an *Eco*RI site within ORF4 as described below (GenBank accession no. DQ418487). An examination of the genome sequences of *B. pseudomallei *that are available on GenBank showed that 8 of the 10 strains possess the same modification at this position as isolate 112. Among all of the *B. pseudomallei *isolates, including 112 and 204, there are between 4 and 19 nucleotide substitutions within this 1472 bp region. Furthermore, isolates that possess the "A" substitution at position 1119 have substantial modifications to sequences flanking this region of the genome (Figure 1), including the presence of a 450 bp insertion (between nucleotides 9565 and 9566 of GenBank accession no. DQ418486) encoding a product with homology to a bacteriophage phiE125 gp30 protein (GenBank accession no. AF447491), and ~650 bp sequence homologous to mutator type transposases from a wide variety of bacterial species, including *Ralstonia solanacearum*, *Pseudomonas syringae*, and *B. thailandensis*. The transposase is probably inactive because of the organization of the sequence, which includes at least 4 stop codons and incomplete reading frames.

**Figure 9:**
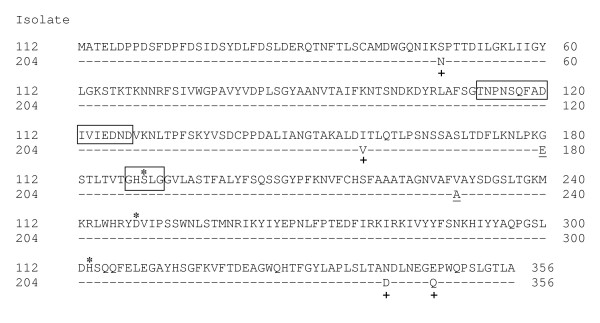
Alignment of lipases of *B. pseudomallei *isolates 112 and 204. Boxes enclose the "lid" motif (residues 112–127) and serine active site (residues 187–191). Asterisks mark the catalytic triad of serine, aspartic acid and histidine. +: conservative amino acid substitutions. Non-conservative amino acid substitutions are underlined. The 6 substitutions that are marked were also present in many of the *B. pseudomallei *genome sequences of 10 isolates that are available in GenBank: S47N: 10 isolates; I157V: 10 isolates; G180E: 5 isolates; V229A: 9 isolates; N340D: 4 isolates; E346Q: 3 isolates. Other substitutions were also found occasionally: S252F: 1 isolate; D341A: 1 isolate; V249L: 1 isolate.

Among the isolates of *B. pseudomallei *that were examined up to 16 nucleotide substitutions fall within ORF4 and although most of these, including the "A" for "G" substitution which introduces the *Eco*RI site described above, do not alter the predicted primary translation there are from 2–4 conservative amino acid substitutions. Non-conservative substitutions which are present, G180E (6/12 isolates) and V229A (10/12 isolates), flank the serine active site and may have functional consequences (Figure 9). Lipases hydrolyze long-chain acyl-triglycerides, a process that usually involves activation of the enzyme at the lipid/water interface, and are known to facilitate the colonisation of plants by fungi and bacteria [47]. The active site is hidden until activation occurs when the "lid" covering the active site opens, exposes the active site catalytic triad, S-D-H, and allows access to the substrate. Mutating specific residues flanking the active site can affect this process, for example by altering the enantiomeric selectivity of *B. cepacia *lipase [48]. *B. pseudomallei *is often associated with disease among workers in rice fields in Thailand and it has been suggested that it may interact closely with plants by using a plant pathogen-like TTS gene cluster [49-51]. In this case, the delivery of lipases could play an essential role in the colonization of plants by *B. pseudomallei*.

Studies investigating the molecular diversity of different isolates of *B. pseudomallei *using a wide variety of methods have demonstrated that although distinct groupings can be defined substantial diversity exists and many isolates do not fall into readily definable groupings or clinical outcomes [3]. Recently, whole genome DNA and RNA spotted arrays have been used to examine large scale genomic variation among different *Burkholderia *species [33,34]. This work has demonstrated the close relationship that exists between the non-pathogenic environmental saprophyte *B. thailandensis*, the obligate equine parasite *B. mallei *and the opportunistic pathogen *B. pseudomallei *and particularly between *B. mallei *and *B. pseudomallei*, supporting work by Godoy *et al*. [52] who considered *B. mallei *to be a clone of *B. pseudomallei *rather than a separate species. Kim *et al*. [33] showed that the expression of a substantial number of genes is highly conserved between all 3 species and suggested that the evolution of small sets of genes rather than large scale acquisitions and losses define the different adaptations of each species. On the other hand, Ong *et al*. [34] proposed that the same 3 species may have diverged through the loss of large chromosomal segments and defined 3 distinct molecular subtypes of *B. pseudomallei *on the basis of regions of difference, constituting areas of DNA recombination that are often characterized by open reading frames (ORFs) encoding products related to phage proteins, transposase or integrase proteins. However, apart from rRNA genes [53] few studies have assessed the extent of variation at specific loci in *B. pseudomallei*.

The diversity of organisms in tropical countries in which melioidosis is endemic and the frequent interactions that may be possible in that environment and within the human host could create opportunities for genetic change [54]. However, a mutation that confers a selective advantage in one environment is unlikely to be a benefit in all environments [55]. Winstanley *et al*. [56] assessed the extent of variation in the flagellin genes of *B. pseudomallei *by comparing the sequences that were obtained from environmental and clinical isolates of the pathogen. The sequences showed either 100% identity or differed by a single nucleotide demonstrating a remarkably conservative nature in these genes. Here, we found a consistent variation in a small region of the *B. pseudomallei *genome downstream from the *mrgRS *locus involving up to 19 nucleotide substitutions including one which changes GA**G**TTC to GA**A**TTC, the recognition sequence for the restriction enzyme *Eco*RI. The consequent restriction fragment length polymorphism (RFLP) was shown to separate *B. pseudomallei *isolates from clinical and environmental sources and from spatially and temporally separate origins, into two distinct groups of almost equal proportions. There was no apparent association between the source of the isolates, the date and place of isolation, and the RFLP pattern. Nevertheless, the substitutions represent a stable genetic modification since they have been retained over time. Furthermore, a search of the genome sequences for *B. pseudomallei *isolates that are available on GenBank revealed the presence of the same modification in many of the isolates. While it is possible that these sequence differences may have occurred at random, the almost equal distribution of the polymorphism among the isolates, the presence of a 450 bp insertion and the close proximity of an inactive transposase suggest that it is much more likely that some process of selection has occurred. For example, differences in primary or secondary host factors may act to select isolated subpopulations according to transmission route or host immune genotypes and play a role in the selection for a mutation or recombination event arising within a bacterial population [57,58]. How this may have occurred in the isolates we examined here is not known. Ong *et al*. [34] described *B. pseudomallei *strain subtypes isolated from different primary hosts, pigs (G1 strains) and melioidosis patients (G2 strains), and so we propose that a selective process involving primary hosts or other vectors, including amoebae, fungi or plants [59-61], may have played a role.

## Conclusion

This work was initiated and completed well before the *B. pseudomallei *genome sequence was published. Although the completion of the *B. pseudomallei *genome sequence has provided a huge body of annotated nucleotide sequence data and an examination of this data revealed at least 33 potential two-component systems in the sequence annotations, very few of the putative genes have been specifically confirmed to be expressed at the protein level in different *B. pseudomallei *strains. In fact, there are substantial discrepancies in the gene and protein annotations for the completed genome sequences of *B. pseudomallei *isolates K96243 and 1710b that are available on the GenBank database. Our work demonstrates that *mrgR *and *mrgS *are present and expressed as protein in a variety of *B. pseudomallei *isolates and that the expression of the locus is temperature-regulated which is an essential step that must be completed before pursuing more complex studies aimed at understanding the biological function and pathogenic significance of the *mrgRS *locus. A similar approach will be necessary to prove the specific protein expression of each of the putative genes that have been identified in the *B. pseudomallei *genome rather than simply assuming that all open reading frames that are annotated in the nucleotide sequence of the genome are expressed as the conceptually translated protein.

Because of its' nutritional and environmental adaptability, *B. pseudomallei *has the potential to spread to areas of the world outside southeast Asia and northern Australia. Two-component transduction systems are a major mechanism by which bacteria regulate adaptive responses to environmental stimuli [18]. Although there have been extensive studies of the biology of *B. pseudomallei*, very few of these have investigated the influence of environmental factors on gene expression and the adaptive responses of the bacterium. We have identified and characterized a two-component transduction system from *B. pseudomallei *that is present and expressed in a variety of strains of the bacterium, encoding proteins that are most similar to the regulators of capsular synthesis in other bacteria. In *B. pseudomallei*, the expression of these proteins is regulated in response to temperature rather than pH or salinity and they are more strongly expressed at temperatures found in mammalian and avian hosts. The recognition of MrgR by antibodies in convalescent sera from melioidosis patients suggests that the *mrgRS *locus may have a role in regulating adaptive responses during the course of infection. Because the expression of the genes is increased at higher temperatures then the adaptive responses they control may be particularly important during the initial phases of infection.

It has been proposed that *B. mallei*, *B. pseudomallei*, and *B. thailandensis *represent 3 states of ecological niche adaptation, namely, obligate pathogen, opportunistic pathogen, and saprophyte, respectively [33]. The 3 species are very closely related and the mechanisms of evolutionary divergence are subject to debate [33,34,52]. The absence of the *mrgRS *locus and downstream flanking region from the genome of *B. thailandensis *shows that it is not essential for a non-pathogenic existence. Because of the similarities in gene organization and structure with other signal transduction systems involved in phase change and the regulation of virulence gene expression, it is possible that *mrgS *and the downstream ORFs may have a similar role in adaptive responses of *B. pseudomallei*, which in the case of *mrgS *is regulated by changes in temperature. The absence of this region in *B. mallei *suggests that this function is no longer required but *mrgR *may still have a regulatory role.

### Nucleotide sequence accession numbers

[GenBank:DQ418486, GenBank:DQ418487]

## Methods

### Bacterial strains and growth conditions

Bacterial strains used in this study are listed in Table 1. All strains of *Burkholderia *species were grown at 37°C, except *B. plantarii *and *B. vandii *which were grown at 25°C, on Luria-Bertani (LB) agar or broth, statically. *Pseudomonas aeruginosa *and *Pseudomonas fluorescens *were grown on nutrient agar at 37°C. The identity of *B. pseudomallei *was confirmed by the API 20NE biochemical test [62]. For expression studies, 10 ml LB broths containing NaCl (0.15, 0.5 or 2.2% w/v) were adjusted to pH 5, 6.8 or 8 and a standardized inoculum of 10^7 ^cells was added and incubated for 48 h at 25, 37 or 42°C. *E. coli *strains were cultured on LB agar or broth [63] and when required, culture media were supplemented with IPTG (100 μM), X-gal (100 μg ml^-1^), ampicillin (100 μg ml^-1^), chloramphenicol (25 μg ml^-1^) or kanamycin (25 μg ml^-1^).

### DNA manipulations

Restriction enzyme digestions, DNA blunting and kinasing, DNA ligation, bacterial transformation and DNA hybridisation were performed according to standard methods [63]. Plasmid DNA was isolated using the Miniprep plasmid DNA extraction kit (BioRad), lambda DNA was purified using the Wizard λ prep DNA purification kit (Promega) and DNA fragments, PCR products and ligation mixtures were recovered using the Prep-a-gene DNA purification kit (BioRad). Genomic DNA was extracted using the Puregene D-6000 DNA isolation kit (Gentra Systems) and quantified by comparison with lambda DNA standards (Sigma Aldrich) using digital images of 1% agarose gels stained with ethidium bromide.

### Preparation of oligonucleotide probes

PCR primers (Sigma Genosys), 5'-GATTTCACGATGCATCAGGCGAAC-3' and 5'-TTCTGGATCGCCGCGATGTCCGTG-3', were derived from the sequences of response regulator genes from other bacterial species to amplify a 377 bp sequence from *B. pseudomallei *204 genomic DNA. The PCR reaction contained 1 unit *Taq *DNA polymerase in reaction buffer (Roche), 1.5 mM MgCl_2_, 10 pmol of each primer, 0.2 mM of each dNTP and 10 ng genomic DNA. Reactions were placed at 96°C for 2 min, followed by 35 cycles consisting of 96°C for 30 s, annealing at 45°C (1st cycle), 50°C (2nd and 3rd cycles) and 60°C (remaining 33 cycles) for 30 s, 72°C for 90 s and a final extension at 72°C for 5 min. Amplicons were analysed in 1% agarose gels, purified and subcloned into pUC18 using *E. coli *DH5α. Cloned DNA was fully sequenced in both directions (MWG Biotech). Following confirmation, the insert was excised from pUC18 by *Eco*RI and *Pst*I digestion, purified by agarose gel electrophoresis and labelled with digoxigenin-11-dUTP (Roche).

For RFLP analysis of 4994 bp of the genome downstream from the *mrgRS *locus, three pairs of PCR primers (Table 2) were designed on the basis of nucleotide sequence data (GenBank accession no. DQ418486) to amplify oligonucleotide probes to sequentially examine 5 contiguous *Eco*RI fragments of the genome. The probes were designated FR1, a 665 bp probe targeting a 1276 bp segment with an internal *Eco*RI site and including the *mrgS *stop codon, FR2, a 1062 bp probe targeting a 1937 bp segment with an internal *Eco*RI site, and FR3, a 1136 bp probe targeting a 1781 bp segment. The PCR reaction contained 1 unit Taq polymerase in reaction buffer (Roche), 1.5 mM MgCl_2_, 24 pmol of each primer, 0.2 mM of each dNTP and 10 ng genomic DNA. Reactions were placed at 96°C for 2 min, followed by 35 cycles of 96°C for 40 s, 65°C for 40 s, 72°C for 90 s and a final extension at 72°C for 5 min. Amplicons were analysed in 1% agarose gels, purified and subcloned into pUC18 using *E. coli *DH5α. Cloned DNA was fully sequenced in both directions (MWG Biotech) and was labelled with digoxigenin-dUTP (Roche).

**Table 2:** PCR primers for preparing oligonucleotide probes FR1, FR2, and FR3

Primer	Sequence 5'-3'
FR1 forward	ATGATGGACGGTTTCCAGTTGCTC
FR1 reverse	AACGTTAAATCAAGTCGCGGGTGG
FR2 forward	AGAGCGCTGTCGCAACTGAATCTG
FR2 reverse	TCGCTTCGCTTGCTGAGAAA
FR3 forward	GGTCCGGGCCAAATATTACGATCC
FR3 reverse	AGCGGAACCAATCCGAACTCACAG

### Construction and screening of genomic DNA libraries

*B. pseudomallei *204 chromosomal DNA libraries were constructed using λGEM-11 and λZAP Express vectors. For λGEM-11, genomic DNA was partially digested with *Sau*3A to yield fragments between 9 and 23 kb which were inserted into the *Bam*HI site and packaged recombinants were plated on *E. coli *LE392 using NZCYM-agarose containing 0.2% maltose (Promega). For λZAP Express, genomic DNA was completely digested with *Bcl*I and ligated into the *Bam*HI site, packaged, and propagated on *E. coli *XL1-Blue. Plasmid DNA, in the form of the kanamycin-resistant phagemid pBK-CMV, containing cloned DNA inserts was excised from λZAP Express using helper phage and *E. coli *XLOLR (Stratagene).

For screening *B. pseudomallei *genomic libraries, phage plaques were overlaid with gridded nitrocellulose filters (Schleicher and Schuell) for 30 min at 4°C. Filters were processed for hybridization with a labelled oligonucleotide probe at high stringency overnight at 68°C according to standard methods [63]. Positive phage plaques were detected with anti-digoxigenin antibody (Roche).

### DNA sequencing and sequence analysis of cloned products

Both strands of cloned DNA were sequenced (MWG Biotech) using either M13 universal and reverse primers or custom synthesized primers (Sigma Genosys). Gene sequences have been deposited in the GenBank database (GenBank accession nos. DQ418486, DQ418487). Gene promoter predictions were made using software provided by Berkeley Drosophila Genome Project [64]. Homology and conserved domain searches were conducted using BLAST software provided by NCBI [65]. Software provided by the Swiss Institute of Bioinformatics [66] and Institut Pasteur [67] was used to analyse secondary structure (PHDtopology), hydrophobicity (Pepwindow), helix-turn-helix motifs (Helixturnhelix), and transmembrane regions (TopPRED2, DAS). Multiple amino acid sequence alignments were performed using MultAlin software [68]. SecretomeP 2.0 [44] was used to predict non-classical protein secretion [69].

### Southern blotting

Genomic DNA (3 μg) was completely digested with *Eco*RI and the fragments separated in 1% agarose gels in TBE buffer, depurinated, denatured and neutralized using standard methods [63]. DNA was capillary blotted onto positively charged nylon membranes (Amersham Biosciences) and fixed to the membrane using UV irradiation. The blots were prehybridized, hybridized with the labelled oligonucleotide probe and developed, according to standard methods [63].

### Synthetic peptides and production of affinity purified antibodies

Synthetic peptides consisting of 9 amino acid residues, DTNVDLINC and RKFYSLESN, corresponding to amino acids 206 to 214 located toward the C-terminus of MrgR and amino acids 14 to 22 located in the N-terminus of MrgS, respectively, were synthesized, conjugated to keyhole limpet hemocyanin (KLH) and used to immunize rabbits (Bethyl Laboratories). To facilitate coupling to maleimide-activated KLH a glycine residue was attached to the C-terminus of the MrgR oligopeptide and a cysteine residue was attached to the N-terminus of the MrgS oligopeptide. Antibodies were purified from the hyperimmune serum of immunized rabbits by affinity chromatography using a peptide-Sepharose matrix, filter sterilized and stored at 4°C. The affinity purified antibodies, anti-MrgR and anti-MrgS, were tested for antigen recognition and specificity as described in Additional file 1, Additional file 2 and Additional file 3.

### Preparation of *B. pseudomallei *protein samples and Western blotting

Cells from 1 ml aliquots of *B. pseudomallei *cultures that had been prepared as described above were centrifuged (4,000 g, 10 min, 4°C), washed in PBS and resuspended in lysis buffer (0.75 M Tris-HCl pH 8.8 containing 0.2% SDS). *B. pseudomallei *broth culture supernatants containing extracellular products (ECPs) were centrifuged (15,000 g, 10 min, 4°C) and the supernatant was concentrated 250-fold using Amicon Minicon Miniplus units, 10 K nominal mw cutoff (Millipore). The total protein concentration of cell lysates and ECPs was determined using a protein assay kit (Bio-Rad). Lipase activity in the concentrated supernatants was determined by comparison with uninoculated broth using the fluorogenic substrate 4-methylumbelliferyl oleate (Sigma). For each sample, equivalent amounts of *B. pseudomallei *whole cell lysate (5 μg) or ECPs (2 μg), were diluted in Laemmli buffer and separated on 12% polyacrylamide-SDS gels. Proteins were electrotransferred to nitrocellulose membranes under standard conditions [70]. Membranes were blocked for 2 h in PBS containing 1% casein, incubated overnight with the primary antibody diluted in the blocking solution, washed 3 × 10 min, and then incubated for 2 h at room temperature in secondary antibody conjugated to horseradish peroxidase (Dako A/S). A list of the primary and secondary antibodies and dilutions that were used is provided in Table 3.

**Table 3:** Dilutions of primary and secondary antibodies for Western blotting

**Primary Antibody**	**Dilution**	**Secondary Antibody**	**Dilution**	**Source**
Anti-MrgR	1:500	Swine Anti-rabbit IgG	1:2000	Bethyl Labs
Anti-MrgS	1:300	Swine Anti-rabbit IgG	1:1000	Bethyl Labs
Anti-MBP	1:1000	Swine Anti-rabbit IgG	1:2000	NEB
Convalescent sera pooled from 6 melioidosis patients	1:300	Rabbit Anti-human IgG	1:1000	Ty Pitt, HPA, London

## Authors' contributions

MEM constructed and screened DNA libraries, prepared oligonucleotide probes, performed Southern blotting and Western blotting, participated in the analysis and interpretation of data, co-drafted and co-wrote the manuscript. THG led the conception and design of the study, co-drafted and co-wrote the manuscript, performed DNA manipulations, participated in the analysis and interpretation of data, and annotated the sequences. DABD initiated the melioidosis studies, obtained bacterial isolates, assisted in the interpretation of data, and contributed to writing the manuscript. MLG assisted with the conception and design of the study, cultured bacterial isolates, performed DNA extraction, participated in the interpretation of data, and contributed to writing the manuscript. All authors read and approved the final manuscript.

## Supplementary Material

Additional File 1 Antibody specificity. Methods for verifying antibody specificity; Construction and expression of recombinant fusion proteins; Characterisation of antibodies recognising MrgR and MrgS; Figure legends for Additional Figures Click here for file

Additional File 4 Figure S3. Western blot showing the recognition of *B. pseudomallei *cellular components from 6 isolates by antibodies in convalescent sera pooled from 6 melioidosis patients. Click here for file

Additional File 2 Figure S1. Western blot showing the recognition of MBP-MrgR fusion protein by affinity purified antibodies, anti-MrgR. Click here for file

Additional File 3 Figure S2. Western blot showing the recognition of MBP-MrgS fusion protein by affinity purified antibodies, anti-MrgS. Click here for file
