# Supplementary material for: Characterization of the mrgRS locus of the opportunistic pathogen Burkholderia pseudomallei: temperature regulates the expression of a two-component signal transduction system
Source: BMC Microbiol. 2006 Aug 7;6:70. doi: 10.1186/1471-2180-6-70 (PMC1557856; doi:10.1186/1471-2180-6-70)
Supplement: Additional File 1 — Antibody specificity. Methods for verifying antibody specificity; Construction and expression of recombinant fusion proteins; Characterisation of antibodies recognising MrgR and MrgS; Figure legends for Additional Figures [file 1471-2180-6-70-S1.doc]

**Antibody specificity**

**Construction and expression of recombinant fusion proteins**

Translational fusions of *malE*, encoding maltose-binding protein (MBP), and *mrgR* (*malE-mrgR*) or *mrgS* (*malE-mrgS*) were constructed using the pMAL-C2 expression vector (New England Biolabs, NEB). For *malE-mrgR*, a 701 bp *Pst*I/*Nsi*I fragment, consisting of 472 bp of the *mrgR* coding region and 229 bp of the 3’ downstream region, was inserted into the *Pst*I site of pMAL-C2. For *malE-mrgS*, a 753 bp sequence spanning the *mrgS* start codon from 48 nucleotides upstream to 702 nucleotides downstream was amplified by PCR under the above conditions using forward primer 5'-CTCGGAATTCCACCAACGTCGATCTCATCAACTG-3', containing an added *Eco*RI site, and reverse primer 5'-GTGAAGATCCACGCTCGTCAAGACGAGCGCGTAG-3', containing an added *Bst*YI site. The amplicon was purified and ligated into pMAL-C2 that had been digested with *Eco*RI and *Bam*HI. The stop codon, encoded by the vector, is 174 nucleotides downstream from the *mrgS* insert. The correct orientation of insert DNA was verified by restriction analysis and DNA sequencing. Overexpression and purification of the fused proteins were performed according to the NEB protocol. The total protein concentration of cell extracts was determined using a protein assay kit (Bio-Rad).

**Characterisation of antibodies recognising MrgR and MrgS**

Equivalent amounts of each sample (3 μg protein) were diluted in Laemmli buffer, separated on 12% polyacrylamide-SDS gels and electrotransferred to nitrocellulose membranes as described above. Affinity purified antibodies raised against peptide sequences unique to MrgR (anti-MrgR) or MrgS (anti-MrgS), and antibodies recognising MBP (anti-MBP) were used to probe Western blots of cell lysates of recombinant *E. coli* expressing MBP-MrgR and MBP-MrgS. The translational fusion of *malE* with *mrgR* links maltose binding protein (MBP) at the C-terminus (43.7 kDa) with 157 residues of MrgR (16.9 kDa) to give a product of 60.6 kDa. Anti-MrgR and anti-MBP stained a major band of ~ 60 kDa present in soluble cell extracts following IPTG induction. A few less prominent bands of a smaller size were evident which most likely represent degradation products of the fusion protein (Figure S1; see Additional file 2). A weakly stained band ~ 60 kDa was also present in uninduced cell extracts despite repression of the *tac* promoter before induction. The translational fusion of *malE* with *mrgS* links MBP (42.9 kDa) with 249 amino acids of the N-terminus of MrgS (27.6 kDa) and an additional 58 amino acids (6.2 kDa) encoded by the vector to yield a product of 76.7 kDa. Anti-MrgS and anti-MBP reacted strongly with MBP-MrgS fusion protein, and recognized a major immunoreactive band of ~ 75 kDa that was mainly present in an insoluble form (Figure S2; see Additional file 3). The specificity of anti-peptide antibodies was determined by incubating each primary antibody with its cognate peptide or with an irrelevant peptide in a 1:15 molar ratio for 1 h at 37°C then overnight at 4°C with agitation, prior to use. Preincubating anti-MrgR and anti-MrgS with their cognate immunising peptide completely blocked specific binding whereas preincubation with an irrelevant peptide had no effect. No bands were detected when the primary antibody was omitted from the experiment.

**Figure S1.** Western blot showing the recognition of MBP-MrgR fusion protein by affinity purified antibodies, anti-MrgR. Lane 1: insoluble matter, lane 2: crude cell extract, lane 3: amylose resin purified cell extract, lane 4: cells induced with IPTG for 3 h, lane 5: uninduced cell extract. Lane M: molecular weight markers indicated in kilodaltons (kDa). Arrows indicate the position of the 60.6 kDa MBP-MrgR fusion protein.

**Figure S2.** Western blot showing the recognition of MBP-MrgS fusion protein by affinity purified antibodies, anti-MrgS. Lane 1: insoluble matter, lane 2: crude cell extract, lane 3: amylose resin purified cell extract; lane 4: cells induced with IPTG for 3 h. Lane M: molecular weight markers indicated in kilodaltons (kDa). Arrow indicates the position of the 76.7 kDa MBP-MrgS fusion protein.

**Figure S3.** Western blot showing the recognition of *B. pseudomallei* cellular components from 6 isolates by antibodies in convalescent sera pooled from 6 melioidosis patients. The isolate number is indicated above each lane. Lane M: molecular weight markers indicated in kilodaltons (kDa). See Table 1 for isolate details.
